# Editorial: Innovations in surgical oncology

**DOI:** 10.3389/fonc.2023.1257762

**Published:** 2023-08-09

**Authors:** Beatrice Aramini, Valentina Masciale, Jeroen L. A. van Vugt

**Affiliations:** ^1^ Division of Thoracic Surgery, Department of Medical and Surgical Sciences (DIMEC) of the Alma Mater Studiorum, University of Bologna, Giovanni Battista Morgagni—Luigi Pierantoni Hospital, Forlì, Italy; ^2^ Division of Oncology, Laboratory of Cellular Therapy, Department of Medical and Surgical Sciences for Children & Adults, University-Hospital of Modena and Reggio Emilia, Modena, Italy; ^3^ Department of Surgery, Erasmus Medical Center (MC) University Medical Center, Rotterdam, Netherlands

**Keywords:** innovation, surgical technique, training, surgical simulators, minimally invasive & robotic surgery

Innovation describes the continuous process of developing and defining new surgical techniques (1, 2). In recent years, the increased introduction of minimally invasive surgical (MIS) approaches has been achieved for solid malignant tumor removal (3–5). The importance of creating new MIS approaches for curing cancer with the benefit of reduced hospital length of stay, less pain, and rapid recovery has motivated innovators to implement robotic surgery (6, 7). This is one of the reasons why innovative engineering solutions have been adopted, that is, to overcome the challenges of these new approaches, decrease costs, and help surgeons achieve the most effective results and clinical outcomes, improving the quality of life of the patients (8, 9). For example, robots and medical simulators have successfully addressed the limitations and revolutionized minimal surgical access (10, 11). The introduction of robots into operating rooms has resulted in improvements in the surgeon’s control and visual field (12). Additional benefits have been noted, even for the patient: less tissue damage, shortened hospitalization time to an average of 3–4 days, decreased psychological impact on the patient, reduction of infection risk with the MIS approach, reduction of unwanted surgical complications (e.g., vessel sectioning and nerve damage), and fewer assistants in the operating room (13, 14).

Furthermore, training using surgical simulators offers several benefits and advantages primarily for future surgeons (15). These simulators can be used as a wet laboratory, with a reduced “human cost” considering potential adverse patient outcomes, and surgeons-in-training can learn in a relaxed environment (16). In addition, the progressive development of simulators improves learning approaches, which involve novel methods that are different from the traditional methods. However, while the importance of these new approaches to improving the learning curve of new surgeons’ is an attractive and acceptable adjunct to surgical curricula, the simulators cannot replace the experiences of surgical preceptors (17). The recent establishment of simulation programs in all surgical fields is beneficial for future surgical training, improving patient care and providing surgeons with the opportunity to overcome limitations without anxiety, which is generally considered the norm during the surgical maturation progress (18). The goals of emerging companies have been changed by these enthusiastic approaches, with the new focuses being to provide solutions to overcome the electro-mechanical limitations of the current robotic surgical systems and to build new surgical simulators to address some of the obstacles faced when performing open surgical procedures (19, 20).

Advances in surgery have focused on minimizing the invasiveness of surgical procedures, and a significant paradigm shift has occurred for some procedures in which surgeons no longer directly touch or see the structures on which they operate (21). Advancements in video imaging, endoscopic technology, and instrumentation have made it possible to convert procedures in many surgical specialties from open surgeries to endoscopic procedures (22). Computers and robotics can be used to facilitate complex endoscopic procedures via voice control over the networked operating room, enhancement of dexterity to facilitate microscale operations, and the development of simulator trainers to enhance the learning of new, complex operations (23). Robotic surgery and medical simulators have dramatically altered and improved procedures, and these two methods share several features: both use a mechanized interface that provides visual patient reactions in response to the actions of the health care professional (simulation also includes touch feedback), both use monitors to visualize the progression of the procedure, and both use computer software applications to interface with the health care professional. Both technologies are experiencing rapid adoption, and they are modalities that allow physicians to perform increasingly complex minimally invasive procedures while enhancing patient safety.

It should also be considered that the advent of new molecular diagnostic technologies has improved treatment approaches in multiple branches of medicine, including surgery. The biosocial medicine approach aims to explain how people’s lifestyles impact their health (24, 25). This approach could be revolutionary for medical practice, paving the way for the introduction of biology to patient care. In addition to the biology of the patient, their biography—or lived experience—should be considered; in this way, biosocial medicine offers a unique signature for each patient. It all started with the idea that the patient is the focus of their own clinical care—although statistical and demographic information is also necessary to ensure the provision of precise medicine—and that clinicians should focus on the real person whom they are treating. Currently, progress is rapidly being made in biology, as the world could appreciate in the management of the global COVID-19 pandemic through the advent of the new mRNA vaccines, thanks to advances in genomic and molecular sciences. This progress may represent the basis for the establishment of precision medicine in clinical practice using tailored treatment based on the signature of the patient (26, 27).

This Research Topic includes a broad selection and unique mix of papers from pioneering researchers showing innovations in surgical oncology.

## Author contributions

BA: Conceptualization, Writing – original draft, Writing – review & editing. VM: Supervision, Visualization, Writing – review & editing. JvV: Conceptualization, Visualization, Writing – review & editing.
